# Bidirectional Control of Risk-Seeking Behavior by the Basolateral Amygdala

**DOI:** 10.1523/ENEURO.0168-18.2018

**Published:** 2018-08-02

**Authors:** J. Miguel Cisneros-Franco, Etienne de Villers-Sidani

**Affiliations:** 1 Department of Neurology and Neurosurgery, Montreal Neurological Institute, McGill University, Montreal, QC H3A 2B4, Canada; 2 Centre for Research on Brain, Language, and Music, Montreal, QC H3G 2A8, Canada

**Keywords:** basolateral amygdale, decision-making, optogenetics, punishment, reward, risk

## Significance Statement

Decision-making under risk entails the possibility of simultaneously receiving positive (reward) and negative (punishment) stimuli. To learn in this context, one must integrate conflicting information related to the magnitude of reward and the probability of punishment. Long-term inactivation of the basolateral amygdala (BLA) disrupts this process and increases risky behavior. In a recent study published in the *Journal of Neuroscience*, Orsini et al. (2017) showed that briefly inhibiting the BLA may result in increased or decreased risk-taking behavior, depending on the phase of the decision process in which BLA activity is disrupted. Here, we discuss the results and propose future experiments that could improve our understanding of how the BLA contributes to adaptive learning under risk and uncertainty.

## 

In complex, “real-world” environments, choices made may result in both rewards and adverse outcomes, each associated with different and often-changing probabilities. Decision-making under risk and uncertainty requires sustained attention and constant updating of learned rules to adequately valuate available alternatives. During risky decision-making, rewarding or punishing outcomes encountered following each decision facilitate learning through positive or negative reinforcement, respectively (Wächter et al., 2009). How individuals react to such competing environmental cues has been the focus of numerous studies in decision neuroscience (Preuschoff et al., 2015). These studies show that cultural, social, and genetic factors shape risk preference, although transient internal states, such as mood, fatigue, or recent experience, may also influence an individual’s propensity to risk-taking (Weber and Johnson, 2009).

Located at the crossroads of corticolimbic circuits that mediate reinforcement learning, the basolateral amygdala (BLA) responds to arousing stimuli of both positive and negative valence (Shabel and Janak, 2009) and is necessary for the establishment of reward associations (Baxter and Murray, 2002) and fear conditioning (Krabbe et al., 2017). This functional heterogeneity has made the BLA a prime target to study associative learning using risky decision-making tasks (RDTs), in which rodents have to choose between a small, “safe” food reward and a large, “risky” reward that might be paired with a punishment. Over the last decade, studies using RDTs have shown that pharmacological inactivation and lesions of the BLA increase risk-seeking behavior (Orsini et al., 2015; Piantadosi et al., 2017). However, studies with high temporal resolution allowing trial-by-trial manipulation of BLA activity are lacking. Therefore, it remains unknown whether increased risk-seeking behavior is the result of BLA loss-of-function specifically during the deliberation phase, i.e., in decision-making per se, and/or during the outcome phase, i.e., the associative phase of reward and punishment.

**Figure 1.**
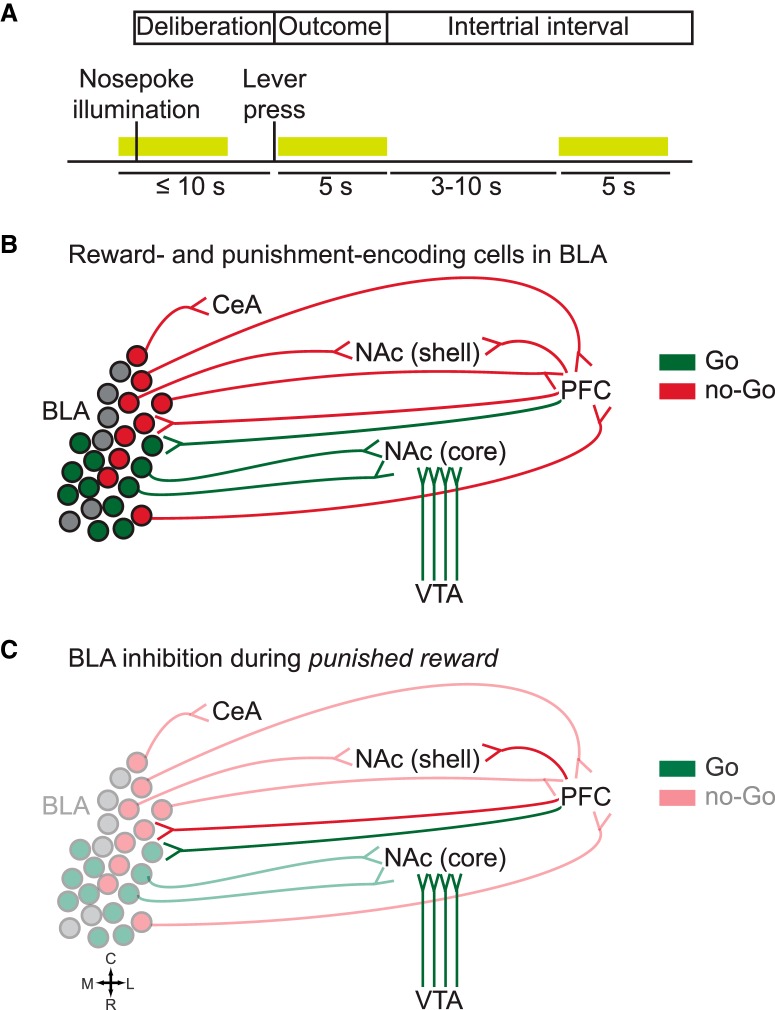
***A***, Experimental paradigm used in Orsini et al. (2017). The RDT was divided in three phases: deliberation, outcome, and intertrial interval. Optogenetic inhibition of the BLA occurred during one of the three phases and lasted ≤5 s. ***B***, Reward (green) and punishment (red) are processed by distinct subpopulations within the BLA. Only major afferent and efferent BLA connections discussed in the text are shown. ***C***, Concurrent delivery of a large reward alongside punishment results in conflicting information, of positive and negative valence, respectively, reaching the BLA. Hypothesis: in absence of *no-Go* inputs from BLA to CeA, NAc shell, and PFC, predominance of *Go* inputs to the NAc (notably from VTA) may bias the animal’s perceived experience toward rewarding stimuli, thus increasing risky choice. R, rostral; C, caudal; M, medial; L, lateral.

In a study published in the *Journal of Neuroscience*, Orsini et al. (2017) addressed this gap in knowledge by using optogenetics to inhibit BLA activity at three different phases of a RDT; namely, deliberation, outcome, and intertrial interval. The deliberation phase corresponded to the ≤10-s period during which rats had to choose between two levers (safe, associated with a small reward, or risky, associated with a large reward that was accompanied by an increasing probability of a footshock). Lever choice marked the beginning of the 5-s outcome period during which reward (always) and punishment (if any) were delivered, followed by a variable intertrial interval that brought each trial’s length to 40 s. Laser delivery occurred during one of the three phases and lasted ≤5 s (Fig. 1*A*).

In the control condition, as expected, the likelihood of choosing the risky reward diminished as the probability of punishment increased. Interestingly, transient BLA inactivation yielded opposite results depending on the phase of the decision-making process at which it occurred. On the one hand, BLA inhibition during the deliberation phase decreased risky choice and resulted in a higher probability of performing a lose-shift, i.e., making a safe choice immediately after punishment. On the other hand, silencing of BLA neurons during delivery of a large, punished outcome, increased risky choice, and decreased the proportion of lose-shift trials.

How to reconcile such disparate results? One possible explanation comes from the fact that optogenetic inhibition reduced overall BLA activity, without cell-type specificity. Because the BLA receives inputs from and sends projections to different cortical and subcortical targets (O’Neill et al., 2018), it is important to consider its functional and anatomic heterogeneity before discussing Orsini et al.’s results.

Inputs reaching the BLA from the prefrontal cortex (PFC) and dopaminergic neurons in the ventral tegmental (VTA) modulate reward sensitivity. Disruption of PFC-to-BLA projections, pharmacological blockade of D_1_ receptors (D_1_R), and D_2_R stimulation all reduce risky choice (St Onge et al., 2012; Larkin et al., 2016). D_1_R stimulation however may increase or decrease risky choice, depending on reward probabilities and individual risk-preferences (Larkin et al., 2016).

Regarding BLA outputs, recent evidence suggests that risk-seeking and risk-avoidance are mediated by distinct neuronal subpopulations within the BLA, which in turn project to segregated brain regions driving opposing outcomes (Namburi et al., 2015; Beyeler et al., 2016, 2018). BLA neurons that synapse in the nucleus accumbens (NAc projectors) are preferentially excited to reward-predictive cues (Beyeler et al., 2016), whereas central amygdala (CeA) projectors and medial PFC (mPFC) projectors are preferentially excited to cues associated with an aversive outcome (Burgos-Robles et al., 2017; Fig. 1*B*). In contrast, neurons projecting to the ventral hippocampus do not show a preference for either positive or negative stimuli (Beyeler et al., 2016). Moreover, a study by the same research group showed that specific activation of BLA NAc projectors and CeA projectors facilitate positive and negative reinforcement learning, respectively (Namburi et al., 2015). Taken together, these results suggest that distinct BLA subpopulations are engaged differently at discrete time-points of the decision-making process. Hence, it is possible that non-specific inhibition of pre- and/or postsynaptic activity within the amygdala might have selective effects depending on the phase of the decision-making process that is disrupted.

## BLA Inhibition during Delivery of Large, Punished Outcome Increases Risky Choice

Delivery of a large reward alongside punishment results in conflicting positive- and negative-valence signals simultaneously reaching the BLA. The increase in risk-seeking behavior reported by Orsini et al. (2017) with BLA inactivation during delivery of a large, punished reward suggests that intact BLA function contributes to the integration of reward magnitude and punishment-related information.

Given the role of BLA NAc-, CeA-, and mPFC-projecting neurons in reinforcement learning, it seems sensible to hypothesize that conflicting outcomes are represented in the BLA by opposing yet complementary activity of these neuronal populations. The increase of risky choice and the reduction in the number of lose-shift trials observed by Orsini et al. (2017) suggests that, in the presence of conflicting inputs, activity of CeA and/or mPFC projectors is more determinant than that of NAc projectors in informing subsequent decisions. If, indeed, discrete BLA subpopulations are preferentially recruited during the outcome phase, inhibition of all projection neurons would have an impact only on the neurons that are normally activated during said phase. This raises the question of why, in the absence of BLA to NAc inputs, risk-seeking behavior is not affected. According to our current understanding of NAc reward circuitry, there are at least two possible, non-mutually exclusive, explanations for this result. The first possible explanation relates to the functional and anatomic subdivisions of the NAc, while the second has to do with other brain structures besides the BLA that feed into the NAc.

First, the BLA projects to both the lateral (“core”) and medial (“shell”) subdivisions of the NAc. A recent study showed that pharmacological inactivation of either BLA or NAc shell during a RDT increased risky behavior, suggesting that both structures suppress punished reward seeking (Piantadosi et al., 2017). In contrast, NAc core inactivation reduced overall responding, even in the absence of any risk, suggesting that NAc core facilitates reward-seeking, independent of motivational conflict (Piantadosi et al., 2017). It is therefore possible that non-specific BLA inactivation affected a BLA-NAc shell circuit responsible for the punishment-induced inhibition of behavior.

Second, the NAc is a major target of dopaminergic VTA neurons, which encode the value of predicted and observed rewards and respond strongly to rewards during the course of learning (Hollerman and Schultz, 1998). Moreover, VTA stimulation following a “risky loss,” i.e., punishment in the absence of reward, increases risk preference (Stopper et al., 2014). Taken together, these observations indicate that BLA silencing during delivery of a large, punished reward might result in or resemble the effect of reduced NAc shell activity, effectively “releasing” the NAc core, which may also respond to strong VTA inputs that bias the animal’s perceived experience toward rewarding stimuli (Fig. 1*C*).

## BLA Inhibition during Deliberation Decreases Risky Choice

The result of decreased risky choice with BLA inactivation during deliberation observed by Orsini et al. (2017) appears to be at odds with previous reports using lesions and pharmacologic inhibition of the BLA (Orsini et al., 2015; Piantadosi et al., 2017). Nevertheless, as mentioned above, said techniques do not offer the time resolution needed to study the role of the BLA during different phases of the RDT, a limitation that was circumvented by the use of optogenetics. Therefore, the experiments conducted by Orsini et al. (2017) demonstrate that previously observed deficits in decision-making following BLA inactivation are the result of BLA loss-of-function that specifically affected the integration of conflicting outcomes and not the deliberative process itself. Given that decreased risk-seeking behavior was elicited exclusively with BLA silencing during the deliberation phase, it remains to be seen which network(s) within the BLA inform decision-making during such a brief period, lasting no more than 5 s in the study by Orsini et al. (2017). A viable approach to answer this question would be the use of optogenetics to identify (“phototag”) BLA subpopulations by simultaneously injecting a Cre-dependent opsin construct in the BLA and a construct carrying Cre recombinase in the structure where the population of interest projects (Beyeler et al., 2018). Should a neuronal BLA subpopulation be identified as key in decision-making during deliberation, this approach could be used in combination with computational models developed for functional neuroimaging to better characterize any observed activity patterns (Prévost et al., 2013). Of particular interest would be to test whether the BLA performs model-based computations, in which the value of actions are updated using a rich representation of the structure of the decision problem, as opposed to purely prediction-error driven model-free algorithms (Corrado and Doya, 2007).

## Concluding Remarks and Future Directions

The main contribution of the study by Orsini et al. (2017) is the demonstration that the BLA plays different roles at different behavioral phases of risky decision-making. While these findings may have implications for the study of impulse control disorders, it should be noted that certain aspects of risky decision-making can be encountered in everyday situations and may have real-life consequences with respect to personal finances. For instance, Knutson et al. (2011) showed that individuals who were better at positive reinforcement learning had more assets, whereas those who were more effective at learning from negative outcomes had less debt. In a subsequent study, the authors found that reduced impulse control, but not cognitive abilities, was the main factor that predicted an individual’s susceptibility to investment fraud (Knutson and Samanez-Larkin, 2014).

Future studies may build on the work of Orsini et al. (2017) to further our understanding of how the BLA contributes to adaptive learning in the context of risk and uncertainty. To this end, we propose two avenues of research going forward. First, investigate if the effects of BLA inhibition vary as a function of individual differences in risk propensity. Although most subjects show a marked bias toward risk aversion, a few seem to prefer risk. Recent studies have shown that risk-seeking rats could be “converted” to risk-averse rats using phasic optogenetic stimulation of D_2_R neurons in the NAc (Zalocusky et al., 2016) or D_2_R agonists infused into the BLA (Larkin et al., 2016). Thus, a priori identification of risk-prone and risk-averse subjects may reveal whether acute BLA inhibition has the same effect on behavior regardless of previously established risk preference.

Second, it remains to be seen whether repeated transient manipulation of BLA activity over an extended period of time will result in significant and long-lasting changes in reward processing. For instance, chronic mPFC stimulation in rats reduces reward-seeking behavior and gives rise to brain-wide activity patterns that predict the onset and severity of anhedonia (Ferenczi et al., 2016). Given the numerous reciprocal connections between the BLA and other brain regions, including the mPFC, we posit that chronic manipulation of BLA activity could similarly change corticolimbic synchrony and alter reward sensitivity. Experiments using intermittent optogenetic or sustained chemogenetic manipulation of BLA activity could be used to test this hypothesis. Going forward, research with a focus on brain networks, rather than on specific isolated structures, may best reveal the mechanisms whereby different behaviors arise from common brain regions.

## Synthesis

Reviewing Editor: Carmen Sandi, Swiss Federal Institute of Technology Decisions are customarily a result of the Reviewing Editor and the peer reviewers coming together and discussing their recommendations until a consensus is reached. When revisions are invited, a fact-based synthesis statement explaining their decision and outlining what is needed to prepare a revision will be listed below.

This Commentary on the report by Orsini et al. published in J. Neurosci. In 2017 is considered relevant and well formulated by an expert reviewer and the editor's own assessment. An increasing number of studies report that the basolateral amygdala is involved in multiple behavioral tasks, including learning, valence, anxiety and decision-making. This commentary provides a good synthesis and interesting insights on the role of the BLA in risky decision making, which will provide neuroscientists with a better understanding of the BLA in this specific behavior, in order to build a more integrative model of BLA function. However, we feel that the manuscript is lacking organization and structure, as well as some important conceptual and experimental clarifications.

Below, there is a detailed account of the points to consider and proposed changes:

Line 35: in the last sentence of this paragraph, the author mention the absence of experiments with temporal control of BLA activity. However, the author do not emphasize the importance of such manipulations. It is necessary to state that it remains unknown whether the BLA is involved in the decision making (deliberation phase) and/or in the associative phase of the reward and punishment.

Line 40: please specify that the « risky » reward is paired with a punishment as soon as you introduce it.

Line 44: It is important to define ‘deliberation’, in terms of time and choice: 10 second while the rats has to choose between two levers (safe and risky). Please also specify when the light inhibition is applied.

Line 65-66: Please define ‘lose-shift performance’. Interesting concepts developed include divergent roles of projection-defined populations of the BLA which could be specifically involved in the deliberating or associative phase (outcome). Indeed, only different projector-populations might be recruited during specific phases of the task, and therefore, inhibition of all projection neurons would have an impact only on the population which is normally activated during that phase. This concept should be explained more clearly.

The work of Burgos-Robles et al., 2017, assessing the role of the BLA-mPFC neurons while rats have to make conflicting choices could also be included in this projection-defined section.

Burgos-Robles, A., Kimchi, E.Y., Izadmehr, E.M., Porzenheim, M.J., Ramos-Guasp, W.A., Nieh, E.H., Felix-Ortiz, A.C., Namburi, P., Leppla, C.A., Presbrey, K.N., et al. (2017). Amygdala inputs to prefrontal cortex guide behavior amid conflicting cues of reward and punishment. Nat. Neurosci. 20, 824-835.

While describing the work of Beyeler et al. 2016, the wording could be improved to more precisely report their findings. Please consider the following change: Line 57: (NAc projectors) are preferentially excited to reward-predictive cues, whereas central amygdala (CeA) projectors are preferentially excited to cues associated with an aversive outcome.

Another possibility is that different inputs to the BLA are active at specific time points of the behavioral task and inhibition of the post-synaptic neurons would then have selective effects. In this context, dopaminergic inputs could be discussed, and (Larkin et al., 2016) should be cited.

Larkin, J.D., Jenni, N.L., and Floresco, S.B. (2016). Modulation of risk/reward decision making by dopaminergic transmission within the basolateral amygdala. Psychopharmacology (Berl.) 233, 121-136.

Finally, when describing three avenues of research, although interesting they are not grounded in the synthesis of the commentary, and feel really disconnected from the rest of the text and from each other.

Inter-individuality is indeed an important factor worth exploring, however, the concept needs to be introduced before.

Similarly, the third avenue is very unclear, and does not relate to any of the work described in the commentary. Moreover, how would DREADDS allow to do patterns of stimulations?

A summary diagram would also help the authors to better organize and communicate their hypotheses and proposed research directions.

Minor Comments________

Line 115: Plays different roles [at different behavioral phases] of risky decision making.

Line 126: ‘it would be interesting to’ should be deleted = First, investigate whether the effects of...

Line 127: as a function ‘OF’ individual differences.
